# Construction and Investigation of MicroRNA-mRNA Regulatory Network of Gastric Cancer with *Helicobacter pylori* Infection

**DOI:** 10.1155/2020/6285987

**Published:** 2020-07-25

**Authors:** Ping Yang, Junjie Liu, Tianci Yang, Lei Zhang, Peiyou Gong, Boqing Li, Xiuzhi Zhou

**Affiliations:** ^1^Department of Pathology, Yantai Yuhuangding Hospital of Qingdao University, Yantai 264000, Shandong Province, China; ^2^School of Basic Medical Sciences, Binzhou Medical University, Yantai 264003, Shandong Province, China; ^3^School of Clinical Medicine, Binzhou Medical University, Yantai 264003, Shandong Province, China; ^4^Department of Infectious Diseases, Yantai Affiliated Hospital of Binzhou Medical University, Yantai 264100, Shandong Province, China; ^5^Department of Medicine, Yantai Yuhuangding Hospital of Qingdao University, Yantai 264000, Shandong Province, China

## Abstract

**Background:**

*Helicobacter pylori* (*H. pylori*) is a common human pathogen, which is closely correlated with gastric cancer (GC). However, the mechanism of *H. pylori*-related GC has not been elucidated. This study aimed to explore the role of *H. pylori* infection in GC and find biomarkers for early diagnosis of *H. pylori*-related GC.

**Methods:**

We identified differentially expressed microRNAs (DEMs) and genes (DEGs) from the Gene Expression Omnibus (GEO) dataset, constructed microRNA-(miRNA-)mRNA expression networks, analyzed the function and signal pathway of cross-genes, analyzed the relations between cross-genes and GC prognosis with the Cancer Genome Atlas (TCGA) data, and verified the expression of cross-genes in patients with *H. pylor*i infection.

**Results:**

22 DEMs and 68 DEGs were identified in GSE197694 and GSE27411 dataset. 16 miRNAs and 509 genes were involved in the expression network, while the cross-genes of the network were mainly enriched in MAP kinase (MAPK) signaling pathway and TGF-beta signaling pathway. Patients with higher expression of hsa-miR-196b-3p, CALML4, or SMAD6 or lower expression of PITX2 or TGFB2 had better outcomes than those with lower expression of hsa-miR-196b-3p, CALML4, or SMAD6 or higher expression of PITX2 or TGFB2 (*P* < 0.05). Patients with *H. pylori* infection had a higher expression of hsa-miR-196b-3p and CALML4 than those without *H. pylori* infection (*P* < 0.05).

**Conclusion:**

The study of miRNA-mRNA expression network would provide molecular support for early diagnosis and treatment of *H. pylori*-related GC.

## 1. Introduction

Gastric cancer (GC) is one of the most common cancers in China, with half a million deaths annually [1]. Because of population growth and life extension, the incidence and mortality rates of GC are increasing [2]. Therefore, it is urgent and important to identify the key genes in its pathogenesis. *Helicobacter pylori* (*H. pylori*) is acknowledged as a class I carcinogen [3], which colonizes in the gastric mucosa and causes chronic gastric, atrophic gastric, and GC [4]. However, how *H. pylori* infection is involved in the pathogenesis and development of GC is unknown [4–6]. Therefore, elucidating the molecular mechanism of *H. pylori*-related GC is of great importance for its early diagnosis and targeted therapy.

MicroRNAs (miRNAs) are 19∼25-nucleotide-long endogenous noncoding RNAs, which negatively regulate the expression of their targets at the posttranscription level and play significant roles in many biological processes, such as proliferation, differentiation, and apoptosis [7–9]. Evidence has been increasing that abnormal miRNAs expression is involved in the pathogenesis and development of many cancers, which suggests the promising biomarkers for early diagnosis and therapy of tumors [5, 10]. Now, a high-throughput platform combined with bioinformatics analysis has become a new way to identify biomarkers of disease [11, 12].

In this study, differentially expressed miRNAs (DEMs) and differentially expressed genes (DEGs) of gastric biopsy with *H. pylori* infection from the Gene Expression Omnibus (GEO) database [13, 14] were identified by R software. After predicting the potential targets of DEMs, we constructed the coexpression network of miRNA-mRNA to identify hub genes. The hub genes were identified using bioinformatics methods including gene ontology (GO) annotation [15] and Kyoto Encyclopedia of Genes and Genomes (KEGG) [16] signal pathway enrichment analysis, while the prognostic value of hub genes was analyzed from the Cancer Genome Atlas (TCGA) database, and the expression of hub genes was confirmed in 69 gastric specimens with or without *H. pylori* infection. We hope this study will provide new information for the molecular mechanism of *H. pylori*-related GC.

## 2. Materials and Methods

### 2.1. Microarray Data

The miRNA and gene expression profiles were obtained from the GEO dataset (https://www.ncbi.nlm.nih.gov/geo/). The screening criteria for GEO were as follows: (1) human gastric mucosa samples with or without *H. pylori* infection; (2) datasets were raw or standardized. The miRNA expression profile (GSE19769) [7] and the gene expression profile (GSE27411) [17] were included in this study. The GSE19769 profile was from the platform of GPL9081 including 10 cases of *H. pylori*-negative and 9 cases of *H. pylor*i-positive gastritis specimen, while the GSE27411 from the platform of GPL6255 containing 6 cases of *H. pylori*-negative and 6 cases of *H. pylori*-positive atrophic gastritis.

### 2.2. Identification of DEMs and DEGs

The significance analysis of DEGs and DEMs in *H. pylori*-negative and -positive samples was performed using the R language software (version 3.6.0, https://www.r-project.org/) and a limma R package. The Benjamini and Hochberg false discovery rate (FDR) method was used to adjust the *P* value to reduce the false-positive risk. The raw data of miRNAs and mRNAs expression were averaged and normalized, and the data of miRNAs were also log2-transformed, while the data with a median expression value of zero or less than zero were removed. *P* value < 0.05 and |log2 fold change (FC)| > 1 were used as the filter threshold for identifying DEGs and DEMs. A pheatmap R package was used for hierarchical clustering analysis and for drawing heat maps of DEGs and DEMs, while a ggplot2 R package was used for drawing volcano plot [13, 14].

### 2.3. Interactive Analysis and Construction Expression Network of miRNA-mRNA

MiRNA-mRNA regulatory networks were constructed to predict the potential *hub genes* of DEMs and DEGs. First, TargetScan (http://www.targetscan.org/vert_72/), miRDB (http://mirdb.org/), PicTar (https://pictar.mdc-berlin.de/), and Miranda (http://miranda.org.uk/) were used to predict targets of DEMs, while different software programs with different algorithms and only genes predicted by at least 3 software programs were selected as the targets of DEMs. The overlapped genes of the targets of DEMs and differential expression genes of GSE27411 dataset were used to construct coexpression networks by Cytoscape software [18].

### 2.4. GO and KEGG Analysis of Cross-Genes

GO analysis is a common approach for gene annotation and gene classification from three aspects of cellular component (CC), biological process (BP), and molecular function (MF) [15]. KEGG is a comprehensive database resource with 17 main databases, which are used to understand advanced gene functions and practical biological systems [16]. The org.Hs.eg.db, clusterProfiler, enrichplot, and ggplot2 R packages were used for GO and KEGG analysis with a cut-off criterion of a *P* value < 0.05 [19].

### 2.5. TCGA Data Processing

The TCGA database is a comprehensive and open database, which contains a variety of human cancer types [20], and was used for validating the relations between the hub genes of the network and the GC prognosis. The inclusion criteria were as follows: (1) the primary site is the stomach; (2) the disease type is the adenomas and adenocarcinomas; (3) the data category is transcriptome profiling; (4) gene expression quantification is used for gene data type, while miRNA expression quantification is used for miRNA data type. Eventually, the mature miRNA expression and clinical data of 452 GC cases (42 normal and 410 having tumors) were downloaded from the TCGA database, and the gene expression and clinical data of 374 GC cases (30 normal and 344 having tumors) were obtained. Survival analysis was performed with a survival R package, while the original data were standardized by the log2 (*x* + 1) method. The prognostic value of cross-genes was determined by Kaplan–Meier analysis, and *P* < 0.05 was considered as a significant difference.

### 2.6. Patient Data

A total of 431 patients, who were confirmed as gastritis or primary GC from March 2019 to October 2019 at the Affiliated Hospital of Binzhou Medical University (Yantai, China) and the Yuhuangding Hospital (Yantai, China), were enrolled in this study. Blood urease was used to detect the *H. pylori* infection. However, 362 patients were excluded because of the lacking of accurate classification or detecting of the *H. pylori* infection. Finally, 69 patients, including 18 negative and 51 positive for *H. pylori* infection, were included in this study. The related clinical and pathological characteristics are listed in Table 1. The samples involved in this study have been approved by the ethics society of Binzhou Medical University and by the patients themselves or their families. None of the patients has received prior radiotherapy or chemotherapy.

### 2.7. Detecting DEMs by SYBR Green Real-Time PCR (RT-PCR)

Total RNA was extracted from paraffin-embedded tissue by miRNeasy FFPE Kit (no. 217504, Qiagen, German), and the RNA concentration and purity were measured by NanoDrop® ND-2000, while adjusting the A260/A280 ratio of RNA solution from 1.8 to 2.1. The cDNA synthesis was performed with the miRNA First Strand cDNA Synthesis (tailing reaction) (no. B532451, Sangon, China). The expression of hsa-miR-196b-3p and hsa-miR-196b-5p was detected in the ABI7500 quantitative PCR system (Applied Biosystems, USA) instrument by SYBR Green Premix Ex Taq II (Tli RNase H Plus, Takara, Japan). U6 small nuclear RNA was used as the internal controls. The 20 *μ*l reaction mixture included 10.0 *μ*l SYBR Green Master Mix, 4 *μ*l cDNA template, 0.4 *μ*l ROX Reference Dye, 1.0 *μ*l primer pairs (10 *μ*m), and 4.6 *μ*l deionized water. PCR cycle was performed as follows: initial degeneration for 30 s at 95°C, followed by 40 cycles of 5 s at 95°C and 34 s at 60°C. The relative expression of hsa-miR-196b-3p and hsa-miR-196b-5p was calculated by comparing the cycle threshold (CT) method using the 2^−△△ct^ method with U6 expression according to [21]. The primers of hsa-miR-196b-3p were 5′-TCGACAGCACGACACTGCCTTC-3′ (sense) and 5′- GACACGGACCCACAGACA-3′ (antisense), while the hsa-miR-196b-5p primers were 5′-GCACCAGCGTAGGTAGTTTCC-3′ (sense) and 5′-TATGCTTGTTCTCGTCTCTGTGTC-3′ (antisense).

### 2.8. Detecting Cross-Genes by Immunohistochemistry

Immunohistochemistry (IHC) was performed in paraffin-embedded sections on the basis of the standardized protocol. Briefly, paraffin-embedded sections (2–4 *μ*m) were deparaffinized with xylene and rehydrated in series gradient ethanol (100%, 95%, and 85% for 1 min, respectively) at room temperature. Heat antigen repair was performed in an autoclave (121°C for 1.5 min) in a citrate sodium buffer (0.01 M), and then endogenous peroxidase was blocked using 3% hydrogen peroxide for 10 min. Sections were incubated with anti-human antibody SMAD6 (ab80049, Abcam), TGFB2 (ab53778, Abcam), PITX2 (ab98297, Abcam), CALML4 (A5086, Zhongshan Golden Bridge, Beijing), and NRP1 (ab25998, Abcam) for 1 h at 37°C and then incubated with a rabbit polyclonal antibody for 20 minutes. After dying with diaminobenzidine for 5–10 min at room temperature, sections were sealed with neutral balata, respectively. The sections were evaluated with semiquantitative method. Briefly, more than 400 cells were counted in each section, while some necrotic cells and peripheral-colored cells were elided. More than 10% of cells were nuclear; staining in all cells was defined as protein-positive expression, while less than 10% was protein-negative expression.

### 2.9. Statistical Analysis

All data were expressed as mean ± standard deviation (SD) of 3 independent experiments, and statistical analyses were performed with SPSS 17.0 (SPSS Ins., Chicago, IL, USA). The difference of miRNA and protein expression was analyzed with two-tail unpaired *t*-test with *P* < 0.05 considered as significant difference.

## 3. Results

### 3.1. Identification of DEMs and DEGs

**Figure 1:**
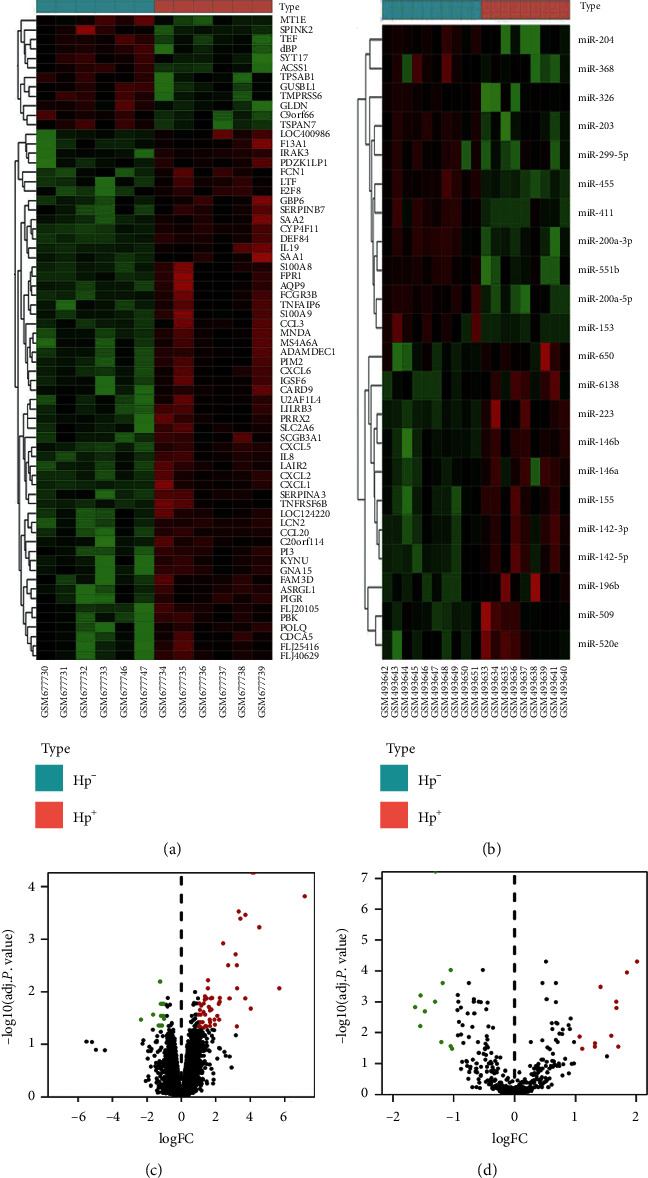
Identification of DEMs and DEGs between *H. pylori*-negative and -positive patients from GEO. Heat map (a) and volcano map (c) for DEGs between *H. pylori*-negative (Hp−) and -positive (Hp+) groups from GSE27411. Heat map (b) and volcano map (d) for DEMs from GSE19769. Red and green colors indicate significant gene overexpression and underexpression, respectively.

The dataset GSE19769 was selected to screen DEMs including 10 *H. pylori*-negative samples and 9 *H. pylori*-positive samples. 470 human miRNAs were analyzed in this dataset, and 22 DEMs have met the filtration criteria of logFC > 1 and *P* value < 0.05, including 11 upregulated and 11 downregulated miRNAs (Table 2). Volcano map and heat map for the hierarchical clustering of the DEMs were carried out by a pheatmap R package (Figures 1(b) and 1(d)). 6 *H. pylori*-negative and 6 *H. pylori*-positive specimens of GSE27411 were analyzed in this study, while there are 18 samples in the dataset. A total of 18187 human mRNAs were expressed, and 68 genes reached the filtration criteria, among which 56 genes showed upregulated and 12 genes showed downregulated expression (Table S1). Volcano map and heat map for the hierarchical clustering of the DEGs were drawn by the pheatmap R package (Figures 1(a) and 1(c)).

### 3.2. Network Construction of the miRNA-mRNA

**Figure 2:**
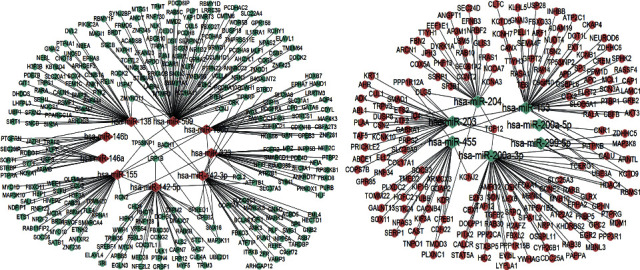
The miRNA-mRNA coexpression network. The diamonds represent aberrant miRNAs, and the circles represent overlapped genes. The red nodes indicate upregulated genes, and the blue indicate downregulated genes. Lines represent an interaction between two genes.

The miRNAs could bind to the 3′ UTR of their targets, resulting in the posttranscriptional suppression of these genes [22, 23]. The biological targets of DEMs were predicted by 4 different software programs, while gene targets of hsa-miR-455, hsa-miR-411, hsa-miR-551b, hsa-miR-509, and hsa-miR-520e were not predicted in the PicTar software. For network construction, the targets were selected in at least 3 databases. The numbers of targets of hsa-miR-455, hsa-miR-223, hsa-miR-200a-5p, hsa-miR-146b, hsa-miR-200a-3p, hsa-miR-155, hsa-miR-411, hsa-miR-551b, hsa-miR-142-3p, hsa-miR-203, hsa-miR-142-5p, hsa-miR-153, hsa-miR-204, hsa-miR-196b, hsa-miR-509, hsa-miR-326, hsa-miR-146a, hsa-miR-299-5p, hsa-miR-520e, and hsa-miR-138 were 88, 43, 9, 18, 131, 60, 36, 2, 65, 89, 65, 151, 69, 48, 189, 25, 18, 18, 20, and 61, respectively (Table S2 and Figures S1 and S2). Intersected genes were obtained with the target genes and all expressed mRNAs in the dataset GSE27411. And all the intersected genes were imported into the Cytoscape software to conduct the miRNAs-mRNA expression network (Figure 2). A total of 16 miRNAs and 509 genes were involved in the network.

### 3.3. GO and KEGG Analysis of Cross-Genes in the Network

To explore the biological functions of the cross-genes of the network, we used an enrichplot and a ggplot2 R package to analyze the GO categories and KEGG signal pathways of the cross-genes. In the BP, the cross-genes were concentrated in outflow tract septum morphogenesis, epithelial cell migration, and endothelial cell migration. In the CC, the cross-genes were enriched in transcription factor complex, synaptic membrane, and adherence junction, but RNA polymerase II proximal promoter sequence-specific DNA binding, proximal promoter sequence-specific DNA binding, and enhancer binding in the MF (Table 3 and Figure S3(A)). KEGG analysis demonstrated that the cross-genes were prominently enriched in the mitogen-activated protein kinase (MAPK) signaling pathway, Ras signaling pathway, and TGF-*β* signaling pathway (Table 4 and Figure S3(B)).

### 3.4. Survival Analysis Verification in TCGA

**Figure 3:**
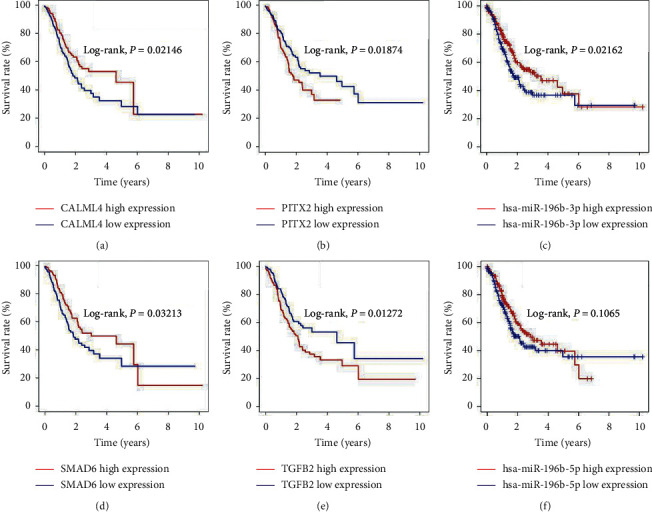
Prognostic value of hub genes for GC patients from TCGA. Overall survival time of GC patients with over- or underexpression of (a) CALML4, (b) PITX2, (c) hsa-miR-196b-3p, (d) SMAD6, (e) TGFB2, and (f) hsa-miR-196b-5p. CALML4: calmodulin-like protein 4; PITX2: paired-like homeodomain 2; SMAD6: SMAD family member 6; TGFB2: transforming growth factor beta 2.

Survival analysis demonstrated that hsa-miR-196b-3p (*P* = 0.02162, Figure 3(c)) was significantly related to the prognosis of GC patients, while hsa-miR-196b-5p was not (*P* = 0.1065, Figure 3(f)), and the patients with lower hsa-miR-196b-3p expression had poor outcomes. CALML4, SMAD6, PITX2, and TGFB2 gene expression were also closely correlated with the GC patients' prognosis. The survival time of GC patients with high expression of CALML4 or SMAD6 was significantly longer than that of patients with low mRNA expression of CALML4 (*P* = 0.02146, Figure 3(a)) or SMAD6 (*P* = 0.03213, Figure 3(d)). In addition, patients with high expression of PITX2 or TGFB2 had a significantly poor prognosis than those with low mRNA expression of PITX2 (*P* = 0.01874, Figure 3(b)) or TGFB2 (*P* = 0.01272, Figure 3(e)).

### 3.5. Validation of miRNA and Cross-Gene Expression

**Figure 4:**
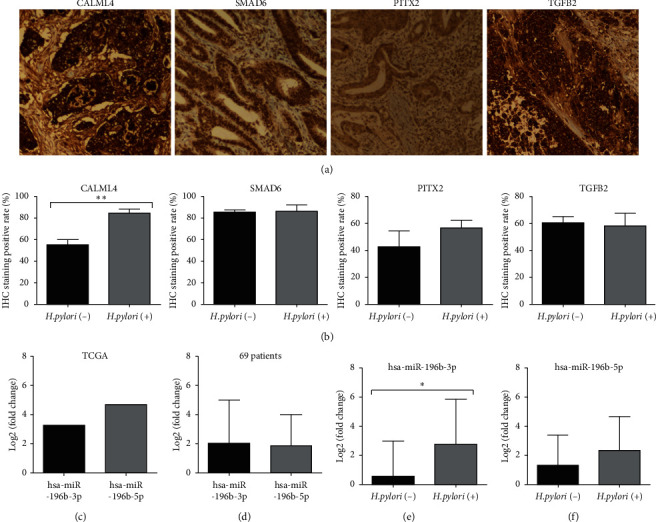
Validation of miRNA and cross-gene expression. (a) Representative immunohistochemical results of CALML4, SMAD6, PITX2, and TGFB2. (b) IHC staining positive rate of CALML4, SMAD6, PITX2, and TGFB2. (c) The expression of hsa-miR-196b-3p and hsa-miR-196b-5p from TCGA database. (d) The expression of hsa-miR-196b-3p and hsa-miR-196b-5p in 69 patients with or without *H. pylori* infection, and the expression level of (e) hsa-miR-196-3p, not (f) hsa-miR-196-3p, in patients with *H. pylori* infection was significantly higher. All tests were independently manipulated at least 3 times, with the conversion of gene expression value to log2 (fold change).

The expression of hsa-miR-196b-3p and hsa-miR-196b-5p was analyzed from the TCGA database to determine its prognostic value of GC. The results revealed that the expression of hsa-miR-196b-5p (log2FC = 3.269, *P* < 0.001) and hsa-miR-196b-3p (log2FC = 4.674216894, *P* < 0.001; Figure 4(c) and Table S3) in GC tissue was significantly higher than that in normal tissue. The expression of hsa-miR-196b-3p and hsa-miR-196b-5p was also detected by qPCR in 69 GC and gastritis patient specimens, which included 18 *H. pylori* negative and 51 positive. The results showed that hsa-miR-196b-3p and hsa-miR-196b-5p were overexpressed in the 69 patients with log2FC values of 2.01665 and 1.8458, respectively (Figure 4(d)). Further analysis showed that the expression level of hsa-miR-196b-3p in *H. pylori*-positive group was significantly higher than that in the negative group (*P* < 0.05, Figure 4(e)); however, there was no significant difference in hsa-miR-196b-5p expression between the *H. pylori-*negative and -positive groups (*P* > 0.05, Figure 4(f)). The protein expression of CALML4, SMAD6, PITX2, and TGFB2 was detected by immunohistochemistry in 69 specimens. The positive rate of CALML4 in *H. pylori*-positive samples (84.3%) was significantly higher than that in negative samples (55%, *P* < 0.01), while there was no significant difference in SMAD6, PITX2, and TGFB2 between the two groups (*P* > 0.05, Figures 4(a) and 4(b)).

## 4. Discussion

Although *H. pylori* infection is closely related to GC, the pathogenesis of *H. pylori-*related GC has not been clarified [7, 24–26]. Therefore, a comprehensive study of the molecular mechanism of *H. pylori-*related GC may be helpful to understand the disease and get better diagnosis and treatment methods. As an important part of bioinformatics, gene expression microarray, which has been widely used in tumor research [27, 28], can analyze the expression of thousands of genes. In this study, the miRNA and mRNA expression profile data of *H. pylori*-infected gastric tissue from GEO database were analyzed, DEMs and DEGs were analyzed with R software, DEMs targets were predicted, miRNA-mRNA expression network was constructed, prognostic value of hub genes for GC was verified, and hub genes expression was detected in the clinical sample. We screened 1 miRNA (hsa-miR-196b-3p) and 4 important nodal genes (CALML4, SMAD6, PITX2, and TGFB2) in *H. pylori*-related GC which can be used as biomarkers for GC prognosis.

miRNA participates in biological, pathological processes and infections by downregulating target expression [29]. The ectopic expression of miRNAs plays an important role in the pathogenesis of multiple cancers, including *H. pylori-*related GC [8, 30]. Several studies have shown that hsa-miR-196b is upregulated in GC and related to the outcomes of GC patients [31, 32], while other research showed that hsa-miR-196b expression in Epstein–Barr virus– (EBV-) positive GC was significantly lower than that in EBV negative [33]. However, the role of hsa-miR-196b in GC, especially in EBV or *H. pylori* infection, is still unknown.

Our study showed that the expression level of hsa-miR-196b in *H. pylori*-positive group was significantly higher than that in *H. pylori*-negative group. Analyzing TCGA data showed that the hsa-miR-196b-3p expression, rather than hsa-miR-196b-5p, could be used as a better biomarker for GC prognosis, which was consistent with the previous report [31]. And we also verified the hsa-miR-196b-3p and hsa-miR-196b-5p expression in clinical samples with or without *H. pylori* infection. The results showed that the expression of hsa-miR-196b-3p in *H. pylori*-positive group was significantly higher than that in the negative group (*P* < 0.05), while there was no significant difference in hsa-miR-196b-5p expression (*P* > 0.05). miRNA target analysis showed that hsa-miR-196b and hsa-miR-326 could regulate the expression of SMAD6 involved in many biological activities through phosphorylation of the TGF-*β* signaling pathway [34, 35]. Then, the results suggested that *H. pylori* may be involved in the pathogenesis of GC with hsa-miR-196b by regulating the expression of many genes and activating the infectious immune pathways. However, the molecular mechanism of hsa-miR-196b in infection and GC needs further experimental study.

Studies have shown that the hsa-miR-200 family, including hsa-miR-200a, participates in the negative feedback loop formed by ZEB1, ZEB2, and SIP1, in which hsa-miR-200 suppresses the expression of ZEB1, ZEB2, and, SIP1 and then downregulates their expression [36, 37]. ZEB2 and SIP1 have also been suggested to inhibit the transcription of cyclin D1 [38]. Some studies showed that, in *H. pylori* infection, the CagA gene can promote transformation from G1 into S/G2 in the cell cycle through activating AP-1 and cAMP, which suggested that hsa-miR-200 was involved in the transformation from gastric epithelial cells to EMT through ZEB loop [39]. Our study showed that hsa-miR-200a-3p showed low expression in *H. pylori*-related GC, and it could inhibit the expression of PITX2 and TGFB2, which participate in the TGF-*β* pathway. Analysis of the relationship between cross-genes and GC prognosis showed that PITX2 and TGFB2 were related to the prognosis of GC. The survival time of GC patients with overexpression of PITX2 and TGFB2 was remarkably shorter than those with underexpression, while hsa-miR-200a-3p had no prognostic value for GC.

Some results have shown that hsa-miR-223 was abnormally overexpressed in GC and was significantly upregulated in *H. pylori*-infected tissues, which could be involved in the pathogenesis of GC by targeting FBXW7 [40]. Some studies showed that hsa-miR-411 showed low expression in GC, and overexpression of hsa-miR-411 in GC cell led to decreasing proliferation and increasing apoptosis [40], while hsa-miR-411 could not be used as an independent predictor of GC prognosis [41]. However, the role of hsa-miR-411 in *H. pylori* infection has not been reported. Our results showed that the expression level of hsa-miR-223 was high in *H. pylori*-positive GC, while that of hsa-miR-411 was low, and both of them can regulate CALML4, which can activate the CGMP-PKG signaling pathway. Besides, patients with overexpression of CALML4 had better outcomes than those with underexpression. However, the mechanism of CALML4 regulated by hsa-miR-223 and hsa-miR-411 in the pathogenesis of *H. pylori-*related GC needs further experimental study.

In conclusion, we constructed a coexpression network of miRNA-mRNA and identified the key genes of hsa-miR-196b, CALML4, PITX2, TGFB2, and SMAD6 in *H. pylori*-related GC, which may provide a new way for the diagnosis and treatment of *H. pylori*-related GC.

## Figures and Tables

**Table 1:** Summary of clinical information of 69 patients.

Characteristics	Patients (*n*)
Age (year)	
≤60	31
>60	38
Sex	
Male	46
Female	23
Histopathological type	
Gastritis	32
Gastric adenocarcinoma	37
*H. pylori* infection	
Negative	18
Positive	51

**Table 2:** Differential expression of miRNAs of *H. pylori*-negative and -positive patients.

miRNA ID	LogFC	*t*	*B*	*P* value	Adj. *P* value	Expression
hsa-miR-455	−1.29961	−13.2889	14.50158	1.32*E* − 10	6.20*E* − 08	Down
hsa-miR-223	2.015725	7.995567	7.036115	2.91*E* − 07	5.02*E* − 05	Up
hsa-miR-200a-5p	−1.04839	−7.29849	5.792449	1.02*E* − 06	9.54*E* − 05	Down
hsa-miR-146b	1.846493	7.098055	5.422075	1.47*E* − 06	0.000115	Up
hsa-miR-200a-3p	−1.18346	−6.46158	4.208209	4.97*E* − 06	0.000249	Down
hsa-miR-155	1.41517	6.230643	3.753807	7.83*E* − 06	0.000335	Up
hsa-miR-411	−1.54782	−5.84129	2.971589	1.71*E* − 05	0.00062	Down
hsa-miR-551b	−1.30788	−5.44908	2.164424	3.85*E* − 05	0.000994	Down
hsa-miR-142-3p	1.67688	5.430155	2.12503	4.00*E* − 05	0.000994	Up
hsa-miR-203	−1.63669	−5.12951	1.494025	7.54*E* − 05	0.001476	Down
hsa-miR-142-5p	1.677953	5.051426	1.32866	8.90*E* − 05	0.001609	Up
hsa-miR-153	−1.47449	−4.88801	0.980864	0.000126	0.002045	Down
hsa-miR-204	−1.55239	−4.24223	−0.41065	0.000513	0.006184	Down
hsa-miR-196b	1.594266	3.80117	−1.36658	0.001355	0.012736	Up
hsa-miR-509	1.069763	3.765869	−1.44288	0.001465	0.013238	Up
hsa-miR-326	−1.20269	−3.53852	−1.93239	0.002417	0.019915	Down
hsa-miR-146a	1.324456	3.446357	−2.1296	0.00296	0.022082	Up
hsa-miR-299-5p	−1.04861	−3.32334	−2.39132	0.003877	0.027607	Down
hsa-miR-520e	1.32247	3.302749	−2.43493	0.004055	0.028448	Up
hsa-miR-650	1.709805	3.294791	−2.45177	0.004127	0.028522	Up
hsa-miR-138	1.114925	3.195115	−2.6619	0.00513	0.033381	Up
hsa-miR-368	−1.02603	−3.19023	−2.67214	0.005185	0.033381	Down

**Table 3:** Top 10 GO functions of the cross-genes in the network.

Category	Gene terms	Count	*P* value
Biological process	GO:0003148, outflow tract septum morphogenesis	10	1.24*E* − 09
	GO:0003151, outflow tract morphogenesis	15	3.98*E* − 09
	GO:0001655, urogenital system development	30	6.76*E* − 09
	GO:0003279, cardiac septum development	17	1.22*E* − 08
	GO:0060411, cardiac septum morphogenesis	13	1.60*E* − 07
	GO:0010632, regulation of epithelial cell migration	26	1.74*E* − 07
	GO:0010631, epithelial cell migration	29	1.75*E* − 07
	GO:0061564, axon development	33	1.77*E* − 07
	GO:0043542, endothelial cell migration	25	1.77*E* − 07
	GO:0090132, epithelium migration	29	2.09*E* − 07

Cellular component	GO:0005667, transcription factor complex	23	4.28*E* − 07
	GO:0097060, synaptic membrane	26	9.71*E* − 06
	GO:0045211, postsynaptic membrane	21	2.70*E* − 05
	GO:0099699, integral component of synaptic membrane	14	3.31*E* − 05
	GO:0005912, adherens junction	28	7.12*E* − 05
	GO:0099240, intrinsic component of synaptic membrane	14	7.62*E* − 05
	GO:0033267, axon part	22	0.000108619
	GO:0000790, nuclear chromatin	20	0.000128584
	GO:0099055, integral component of postsynaptic membrane	11	0.000162968
	GO:0000151, ubiquitin ligase complex	16	0.000198327

Molecular function	GO:0000978, RNA polymerase II proximal promoter sequence-specific DNA binding	38	5.75*E* − 10
	GO:0000987, proximal promoter sequence-specific DNA binding	39	5.92*E* − 10
	GO:0035326, enhancer binding	19	1.24*E* − 09
	GO:0000980, RNA polymerase II distal enhancer sequence-specific DNA binding	15	1.89*E* − 08
	GO:0001158 enhancer sequence-specific DNA binding	16	5.18*E* − 08
	GO:0003682, chromatin binding	34	9.92*E* − 08
	GO:0001228, DNA-binding transcription activator activity, RNA polymerase II-specific	29	2.20*E* − 06
	GO:0004714, transmembrane receptor protein tyrosine kinase activity	9	7.94*E* − 06
	GO:0003712, transcription coregulator activity	32	1.19*E* − 05
	GO:0019199, transmembrane receptor protein kinase activity	10	1.30*E* − 05

**Table 4:** Top 10 KEGG pathways of the cross-genes in the network.

KEGG terms	Count	*P* value
MAPK signaling pathway	25	3.70*E* − 06
Axon guidance	17	3.89*E* − 05
Ras signaling pathway	19	9.06*E* − 05
TGF-beta signaling pathway	11	0.000134809
cGMP-PKG signaling pathway	15	0.000184067
Human cytomegalovirus infection	18	0.000186779
Adrenergic signaling in cardiomyocytes	14	0.00018806
Hepatocellular carcinoma	15	0.000196653
Rap1 signaling pathway	17	0.000244921
ErbB signaling pathway	10	0.000259955
Growth hormone synthesis, secretion, and action	12	0.000281056

## Data Availability

The data used to support the findings of this study are included within the article.

## Abbreviations

*H. pylori*: *Helicobacter pylori*
miRNAs: MicroRNAs
DEMs: Differentially expressed miRNAs
DEGs: Differentially expressed genes
GC: Gastric cancer
GEO: Gene expression omnibus
BP: Biological process
CC: Cellular component
MF: Molecular function
qPCR: Quantitative polymerase chain reaction
KEGG: Kyoto Encyclopedia of Genes and Genomes
GO: Gene ontology
TCGA: The Cancer Genome Atlas
EBV: Epstein–Barr virus
FC: Fold change
SD: Standard deviation
FDR: False discovery rate.

## Supplementary Materials

Supplementary Materials Figure S1: Venn diagram of potential targets of DEMs predicted by 4 software programs. Venn for hsa-miR-455 (A), hsa-miR-223 (B), hsa-miR-200a-5p (C), hsa-miR-146b (D), hsa-miR-200a-3p (E), hsa-miR-155 (F), hsa-miR-411 (G), hsa-miR-551b (H), hsa-miR-142-3p (I), hsa-miR-203 (J), hsa-miR-142-5p (K), and hsa-miR-153 (L). Figure S2: Venn diagram of potential targets of DEMs predicted by 4 software programs. Venn for hsa-miR-204 (A), hsa-miR-196b (B), hsa-miR-509 (C), hsa-miR-326 (D), hsa-miR-146a (E), hsa-miR-299-5p (F), hsa-miR-520e (G), and hsa-miR-138 (H). Figure S3: GO and KEGG function analysis of the cross-genes. Circle diagram of (A) GO clusters and (B) KEGG pathway clusters. Table S1: differential expression genes of *H. pylori*-negative and -positive patients. Table S2: targets of DEMs in the network. Table S3: expression of hsa-miR-196b-3p and hsa-miR-196b-5p in TCGA.
