# Supplementary material for: Construction and Investigation of MicroRNA-mRNA Regulatory Network of Gastric Cancer with Helicobacter pylori Infection
Source: Biochem Res Int. 2020 Jul 25;2020:6285987. doi: 10.1155/2020/6285987 (PMC7410007; doi:10.1155/2020/6285987)
Supplement: Supplementary Materials — Figure S1: Venn diagram of potential targets of DEMs predicted by 4 software programs. Venn for hsa-miR-455 (A), hsa-miR-223 (B), hsa-miR-200a-5p (C), hsa-miR-146b (D), hsa-miR-200a-3p (E), hsa-miR-155 (F), hsa-miR-411 (G), hsa-miR-551b (H), hsa-miR-142-3p (I), hsa-miR-203 (J), hsa-miR-142-5p (K), and hsa-miR-153 (L). Figure S2: Venn diagram of potential targets of DEMs predicted by 4 software programs. Venn for hsa-miR-204 (A), hsa-miR-196b (B), hsa-miR-509 (C), hsa-miR-326 (D), hsa-miR-146a (E), hsa-miR-299-5p (F), hsa-miR-520e (G), and hsa-miR-138 (H). Figure S3: GO and KEGG function analysis of the cross-genes. Circle diagram of (A) GO clusters and (B) KEGG pathway clusters. Table S1: differential expression genes of H. pylori-negative and -positive patients. Table S2: targets of DEMs in the network. Table S3: expression of hsa-miR-196b-3p and hsa-miR-196b-5p in TCGA. [file 6285987.f1.zip › 6285987.f1.docx]

TABLE S1: Differential expression genes of *H.pylori* negative and positive patients

| Gene_ID | LogFC | t | B | *P*Value | adj.*P*Val | Expression |
| --- | --- | --- | --- | --- | --- | --- |
| CYP4F11 | 4.112957 | 13.23567 | 9.733293 | 4.73E-09 | 5.43E-05 | Up |
| CXCL5 | 4.23704 | 12.99005 | 9.5761 | 5.97E-09 | 5.43E-05 | Up |
| DEFB4 | 7.157566 | 11.56057 | 8.564486 | 2.51E-08 | 0.000152 | Up |
| CXCL2 | 3.325672 | 10.67893 | 7.848023 | 6.56E-08 | 0.000298 | Up |
| IL8 | 3.720673 | 10.35322 | 7.563251 | 9.52E-08 | 0.000346 | Up |
| LCN2 | 3.420611 | 10.05495 | 7.292312 | 1.35E-07 | 0.000409 | Up |
| CCL20 | 4.529974 | 9.615894 | 6.874954 | 2.29E-07 | 0.000594 | Up |
| CXCL1 | 2.432357 | 8.959161 | 6.206935 | 5.22E-07 | 0.001186 | Up |
| LAIR2 | 3.145362 | 8.499722 | 5.706333 | 9.54E-07 | 0.001928 | Up |
| SAA2 | 2.730045 | 8.006205 | 5.136074 | 1.87E-06 | 0.00315 | Up |
| LTF | 3.215452 | 7.994767 | 5.122443 | 1.90E-06 | 0.00315 | Up |
| ASRGL1 | 1.550548 | 7.485848 | 4.496287 | 3.94E-06 | 0.005976 | Up |
| TEF | -1.23927 | -7.38323 | 4.365273 | 4.58E-06 | 0.006412 | Down |
| PI3 | 5.6814 | 7.086379 | 3.977038 | 7.14E-06 | 0.008494 | Up |
| S100A8 | 3.234683 | 7.081659 | 3.970753 | 7.19E-06 | 0.008494 | Up |
| IRAK3 | 1.538139 | 7.056196 | 3.936787 | 7.47E-06 | 0.008494 | Up |
| CXCL6 | 1.365178 | 6.741727 | 3.508737 | 1.21E-05 | 0.012242 | Up |
| AQP9 | 2.232812 | 6.633476 | 3.357693 | 1.44E-05 | 0.013052 | Up |
| MNDA | 1.771979 | 6.60249 | 3.314105 | 1.51E-05 | 0.013052 | Up |
| IGSF6 | 1.787686 | 6.569443 | 3.267447 | 1.59E-05 | 0.013126 | Up |
| SERPINA3 | 3.711334 | 6.507429 | 3.179408 | 1.75E-05 | 0.013441 | Up |
| ADAMDEC1 | 1.470828 | 6.469138 | 3.124734 | 1.86E-05 | 0.013441 | Up |
| PIM2 | 1.715581 | 6.45398 | 3.103024 | 1.91E-05 | 0.013441 | Up |
| PIGR | 1.399005 | 6.41611 | 3.048619 | 2.03E-05 | 0.013441 | Up |
| IL19 | 2.797768 | 6.40309 | 3.029861 | 2.07E-05 | 0.013441 | Up |
| TNFRSF6B | 1.349065 | 6.347847 | 2.949957 | 2.26E-05 | 0.014183 | Up |
| PRRX2 | 2.225226 | 6.268296 | 2.834011 | 2.57E-05 | 0.015592 | Up |
| FAM3D | 1.038663 | 6.19041 | 2.719479 | 2.92E-05 | 0.016593 | Up |
| TNFAIP6 | 1.291622 | 6.157535 | 2.670836 | 3.08E-05 | 0.016623 | Up |
| KYNU | 2.168165 | 6.122355 | 2.618585 | 3.26E-05 | 0.016858 | Up |
| C9orf66 | -1.03519 | -6.09822 | 2.582622 | 3.40E-05 | 0.016858 | Down |
| TSPAN7 | -1.18885 | -6.0908 | 2.571544 | 3.44E-05 | 0.016858 | Down |
| SPINK2 | -1.11474 | -6.07604 | 2.549492 | 3.52E-05 | 0.016858 | Down |
| FLJ20105 | 1.151239 | 5.964137 | 2.381067 | 4.24E-05 | 0.018359 | Up |
| FPR1 | 1.595177 | 5.906129 | 2.292951 | 4.67E-05 | 0.019306 | Up |
| C20orf114 | 4.025527 | 5.849972 | 2.207119 | 5.13E-05 | 0.020741 | Up |
| PDZK1IP1 | 1.746548 | 5.809633 | 2.145143 | 5.49E-05 | 0.021715 | Up |
| U2AF1L4 | 1.076033 | 5.792027 | 2.118011 | 5.66E-05 | 0.021894 | Up |
| GNA15 | 1.660556 | 5.763334 | 2.073682 | 5.94E-05 | 0.022503 | Up |
| FCGR3B | 1.289058 | 5.71084 | 1.992234 | 6.49E-05 | 0.024097 | Up |
| CARD9 | 1.001299 | 5.618284 | 1.847532 | 7.60E-05 | 0.026826 | Up |
| E2F8 | 1.122244 | 5.602044 | 1.821999 | 7.82E-05 | 0.026826 | Up |
| DBP | -1.63517 | -5.58091 | 1.788705 | 8.11E-05 | 0.027098 | Down |
| PBK | 1.374776 | 5.574596 | 1.778746 | 8.19E-05 | 0.027098 | Up |
| LOC124220 | 2.084758 | 5.531143 | 1.710024 | 8.83E-05 | 0.02848 | Up |
| SYT17 | -1.05909 | -5.5068 | 1.6714 | 9.21E-05 | 0.02848 | Down |
| GUSBL1 | -1.18185 | -5.50501 | 1.668541 | 9.24E-05 | 0.02848 | Down |
| ACSS1 | -1.01679 | -5.39722 | 1.496316 | 0.000111 | 0.032678 | Down |
| SAA1 | 1.925987 | 5.366392 | 1.446713 | 0.000118 | 0.033931 | Up |
| LOC400986 | 1.646804 | 5.355422 | 1.429027 | 0.00012 | 0.033931 | Up |
| SCGB3A1 | 2.192774 | 5.346785 | 1.415089 | 0.000122 | 0.033931 | Up |
| GLDN | -2.34634 | -5.33132 | 1.390098 | 0.000125 | 0.033931 | Down |
| POLQ | 1.089428 | 5.227436 | 1.221283 | 0.00015 | 0.037713 | Up |
| FCN1 | 1.228984 | 5.219811 | 1.208825 | 0.000152 | 0.037713 | Up |
| LILRB3 | 1.671023 | 5.21963 | 1.20853 | 0.000152 | 0.037713 | Up |
| GBP6 | 1.953245 | 5.120372 | 1.045545 | 0.000181 | 0.042307 | Up |
| TPSAB1 | -1.32145 | -5.08807 | 0.992176 | 0.000192 | 0.043628 | Down |
| MT1E | -1.1254 | -5.0751 | 0.970698 | 0.000197 | 0.043628 | Down |
| TMPRSS6 | -1.2285 | -5.06136 | 0.947939 | 0.000202 | 0.044082 | Down |
| MS4A6A | 1.03852 | 5.033512 | 0.901684 | 0.000212 | 0.044284 | Up |
| FLJ25416 | 1.402461 | 5.020946 | 0.880778 | 0.000217 | 0.044284 | Up |
| FLJ40629 | 1.007559 | 5.004683 | 0.853685 | 0.000223 | 0.044384 | Up |
| S100A9 | 1.660309 | 5.001291 | 0.84803 | 0.000225 | 0.044384 | Up |
| SLC2A6 | 1.454419 | 4.979813 | 0.81218 | 0.000233 | 0.045311 | Up |
| SERPINB7 | 3.214345 | 4.977826 | 0.80886 | 0.000234 | 0.045311 | Up |
| F13A1 | 1.09191 | 4.961326 | 0.781267 | 0.000241 | 0.046186 | Up |
| CCL3 | 1.056503 | 4.950757 | 0.763572 | 0.000246 | 0.046584 | Up |
| CDCA5 | 1.297342 | 4.927722 | 0.72495 | 0.000256 | 0.048061 | Up |

TABLE S2: Targets of DEMs in the network

| miRNA_ID | Target genes |
| --- | --- |
| has-miR-455 | KIF1B; RNF111; NPAS3; DDX3X; CAST; USP3; ESRRG; PTPRS; PPP1R12A; KDM6A; NLK; NXPH1; TJP1; BRD1; HNRNPL; NR4A2; TUBB; AAGAB; CPEB3; C8orf59; CDK14; SEMA4G; MEMO1; DCAF5; CTNND1; TBL1XR1; VCAN; RAB18; GALNT13; FRMD5; ZFPM2; LUC7L3; MYLIP; RANBP3; ADD3; KDSR; RIN2; TRIM33; DOCK9; SECISBP2L; PATL1; IRF2; CDK13; BAZ2B; DYNC1LI2; KDR; TMEM30A; ARMCX3; TSHZ3; S1PR1; R3HDM2; USP9X; HCFC2; ANKRD27; TMEM86A; NCK2; CPEB1; TMEM167A; ZNF516; KCNJ2; NMT2; TNPO1; PTP4A2; PTGFRN; TAF4; SUZ12; KPNA3; PPP2R5A; TPR; PLXNC1; ARHGEF17; CDH22; NRIP1; KIF3A; ARRDC3; TMED2; CACNB4; CDC73; SERP1; GDAP2; STK24; PLXDC2; TOMM22; SOCS3; IPO7; CDKN1B; PHF12; SOX11 |
| has-miR-223 | NFIB; SCN3A; ACSL3; IL6ST; SP3; F3; PDE4D; CALML4; RNF34; FBXW7; RPS6KB1; SLC8A1; HLF; PURB; UBE2A; FGFR2; LMO2; RCN2; CRIM1; NFIA; RASA1; FBXO8; MEF2C; RAB10; ATP1B1; SPRED1; SMARCD1; NUTF2; COPS2; PKNOX1; SLC37A3; KIAA0355; TCERG1; ATP2B1; PTBP2; PRDM1; STIM1; ANKRD17; INPP5B; STK39; MPZ; RAP2A; HHEX |
| has-miR-200a-5p | RBMS1; PRDM10; PITPNB; KIAA1217; CNR1; UBE3A; PSEN1; ZNF644; PPM1E |
| has-miR-146b | USP3; STRBP; PPP1R11; PTPRA; RARB; ROBO1; SEC23IP; KLF7; SYT1; NUMB; SORT1; JAZF1; IRAK1; PTGFRN; PIP5K1B; TRAF6; NOVA1; SAMD8 |
| has-miR-200a-3p | ARNTL; ZFR; MBNL3; SLC25A3; SPAG9; PPP3R1; CDC25A; CYP26B1; RBMS1; ZCCHC3; PITPNB; SON; EPHA7; BAHD1; TNKS2; APBB2; FBXL2; HOXB5; RARB; HIC2; AMPD2; SEMA6A; EVI5L; TTR; PPT2; RBM7; FOXP1; HIPK1; LENG8; CHD9; ARHGEF18; SIRT1; STXBP5; GPHN; RUNX1; MBNL1; PEX5; CCNE2; CALCR; MATR3; ATXN7; TCF12; IRS2; STXBP1; MYRIP; UBE3A; OGT; PDCD4; MAP2K4; CBX1; RAP2C; SLC17A6; YTHDF2; SLC20A1; PPP2CA; PITX2; YPEL5; PGRMC2; TAF12; DR1; SUPT6H; STRNl, ATXN1; PPP2R2A; ELMOD1; DEK; SIAH1; MYH10; PLAG1; TGFB2; TP53INP1; DOLPP1; CDC42; FOXA2; CTNND2; WDFY3; LYPLA1; PRKACB; ZNF644; ATP2A2; EGR2; LHX6; PRKCE; DLC1; PAPPA; NRXN1; CUL3; RAB30; PTPRG; KCNJ2; OSBPL11; YY1; KPNA4; CD47; SLC16A; ELAVL2; SIPA1L2; MDM1;  GATA6; TCERG1; NRP1; E2F3; FKBP5; FOXA1; H2AFZ; JAG1; KPNA3; KHDRBS3; HDAC4; BRMS1L; STAT4; TRHDE; STAT5A; RAB38; PPM1E; HMG20A; BRD3; CDC14A; ATP6V1B2; ASXL1; PCDH8; YWHAG; STAT5B; PPP1R15B; KIF1C; EPHA2; GPC2; SFPQ; KHDRBS2; GLRX; TSC1 |
| has-miR-155 | HIF1A; YWHAZ; UPP2; ACTA1; CSNK1G2; IKBKE; KPNA1; LRP1B; SATB1; BACH1; CSF1R; SP3; AICDA; NR2F2; BRD1; MYB; SMNDC1; MEIS1; SCG2; TLE4; RAB11FIP2; MYO10; YWHAE; FBXO11; FGF7; CARHSP1; CEBPB; OLFML3; RCN2; USP48; PSKH1; WEE1; SPRED1; SOCS1; INPP5D; NDFIP1; ETNK2; SMARCA4; TP53INP1; COPS3; H3F3A; HBP1; RNF123; SALL1; CAB39; MAP3K10; HIVEP2; ZIC3; ZNF236; ANTXR2; SOCS6; SUFU; MYO1D; TOMM20; ETS1; PELI1; ARVCF; FBXO33; KBTBD2; ITK |
| has-miR-411 | EIF4G2; PUM1; KDM6A; FBXL5l; HNRNPH3; LRP12; CDH2; CALML4; C21orf91; CAMSAP1; NUDT4; FRMD4A; RAPGEF2; CACHD1; USP32; SP2; MAP3K1; HSD17B4; GTF2I; CD200; MIB1; SPRY4; UBE2R2; ZNRF3; MAML3; SCD5; PDS5A; DUSP1; DUSP13; ARRDC3; KPNA2; BNIP3; RNF149; RAB21; ELFN1; CPT1A |
| has-miR-551b | ERBB4; GALNTL6 |
| has-miR-142-3p | CRK; ARNTL; ZFR; COPS7A; ASB7; ARHGAP12; PUM1; FMNL2; BACH1; PTPN23; CFL2; IL6ST; RGL2; ITPKB; MARK3; XPO1; GFI1; GNB2; MAP3K11; CPEB2; BCLAF1; ACBD5; STAM; TFG; STRN3; FKBP1A; RARG; RAB1A; ATF7IP; MARCKS; CCNT2; TARDBP; GNAQ; AKT1S1; FBXO3; INPP5A; SLC37A3; EML4; ANK3; TAOK1; ATP2A2; EGR2; IRAK1; ITGAV; SMG1; CLTA; BTBD7; C9orf72; STX12RAC1; HECTD1; TIPARP; SLCO4C1; RERE; GTF2A1; PAFAH1B2; RLF; ADCY9; SP8; LRRC1; VAMP3; ADAMTS3; HMGA2; ZNF217; ROCK2 |
| has-miR-203 | ADK; ETS2; ABL1; ARHGAP12; CCNG1; GLI3; RNF38; COPS7B; SMAD1; IGFBP5; PPP1R12A; NLK; GABRA1; KHDRBS1; TRPV3; STX16; MYEF2; PDE4D; RAP1A; KCTD9; LPP; RNF34; EIF4E; AHR; OSBPL8; DNMT3B; PTPN3; ABCE1; PRPS2; CUL1; EGR3; ATF2; GPR85; TCF12; CDH10; IRS2; PCSK2; MAPK9; MBNL2; SLC4A4; GABARAPL1; PTP4A1; SLC17A6; INSIG1; DGKB; CSN2; MEF2C; AMOT; OVOL1; ELL2; DR1; SLC12A2; SOSTDC1; FMR1; VAV3; TARDBP; XRN2; NBEA; BCL7A; WDFY3; KRT1; CAMTA1; LASP1; KCNK10; AP1G1; TUSC2; CAB39; DLG5; GPC4; MAP4K3; PLAA; TRPS1; GLN1; SLC39A9; PHLDA3; ID4; SOCS6; DLX5; ZNF281; SPARC; TAF5; PLD2; NOVA1; DGKZ; COL17A1; PRICKLE2; ACO2; CITED2; DUSP5 |
| has-miR-142-5p | MBD2; LRP1B; NFE2L2; RHOT1; HERPUD1; EGLN3; SACS; CCNG2; ELAVL4; LRP12; DIO2; HIPK1; CHD9; PTPN4; GRSF1; AHR; RNF128; RPS6KA4; SNX16; CPEB2; TCF12; HDLBP; TRIM3; ZFPM2; VPS54; REV3L; CAMK2A; RCN2; QKI; FBXL3; ALS2; MCL1; UCHL3; ABCA1; ATP1B1; GAS7; RHOA; SP2; MASTL; CUL2; APBB3; MED28; DNAJC7; SRI; STK35; ETV1; FBXO30; STC1; RAB6B; SREBF1; OTX2; UBE2D1; CUL4A; MYCN; TRIM36; SLC36A1; ULK1; ADAMTS1; WWP1; CBX3; CAPN7; CDC37L1; CDK5; MYF5; DCHS1 |
| has-miR-153 | DMD; ARNTL; PHF3; SPHK2; ARF1; ASB7; RAI14; CAP1; CREBBP; GNPDA2; SATB1; SCN3A; NFE2L2; PLCB1; ZDHHC6; RALA; CREM; PPM1D; KLHL5; APBB2; SP3; ZFYVE9; LRP12; PRDM2; LAMP1; KCTD9; KCNB1; CAMKK2; VAMP2; AUTS2; ITPR1; OSBPL6; SEMA4G; SEMA4F; RAB11FIP2; SLC9A6; PTBP1; HEY2; RTN4; MAT2A; CNN3; CANX; CPEB2; ANK1; IRS2; SH3BP4; RABGAP1; SLC4A4; TESK2; ZFPM2l LATS1; NUDT4; RASSF4; APP; FGFR2; FLRT2; CLCN5; KLHL3; TES; HECTD2; FEM1C; FBXL3; ATP2C1; MCL1; APC; RASA1; SYT1; APLP2; DOCK9; TCERG1L; ELL2; CIB2; PPP3CA; RPL22; EPC1; MYCBP; SLCO5A1; SOCS2; KCNH7; PAIP1; TP53INP1; NAV2; NBEA; TPCN1; POU4F1; EXT1; ARID4A; PLEKHM1; USP28; KCNMB2; EFNA3; AMPH; ADAM19; CAMK2G; NEUROD6; CBFB; WRNIP1; SOCS5; KPNA4; ITSN2; GLCE SLC9A9; AKAP6; SMARCD2; HLCS; ADD1; CLTC; JAG1; OTX2; DOT1L; UXS1; PRKAB2; DACH1; ZBTB2; DDIT4; ZCCHC2; RYR2; RNF26; TP53INP2; KLF13; WDR26; GRB2; TAF5; TBX3; PELI1; GALNT3; KCNA6; KCTD5; TTYH3; NR3C2; CKAP4; LAMC1; NEUROD1; AKT3; WWP1; NOVA1; PCDH8; FBXO33; GALNT7; FURIN; ZNRF2; ZDHHC2; GNAI3; RYR3; GFPT2; CITED2; C9orf40; INHBB; EPHA4; ZDHHC1; ROCK2 |
| has-miR-204 | GLIS3; DNM2; MEIS2; ADCY6; EPHA7; ESRRG; CHN2; AKAP1; KHDRBS1; ZNF282; FRAS1; EPHB6; CELSR3; SEC24D; M6PR; MAP1LC3B; NCOA7; ITPR1; EEF1E1; NRBF2; DYRK1A; SIRT1; CREB5; MAPRE2; ANGPT1; DMTF1; SEC61A2; ATF2; TCF12; JPH3; MLLT3; RPS6KC1; AP3M1; EFNB3; CRKL; RAB10; ELAVL3; SLITRK4; LSM5; SPRED1; CCNT2; TMOD3; NBEA; SOX4; SLC37A3; SLC16A6; COX5A; FARP1; RAB22A; DLG5; NR3C1; FBN2; TTYH1; ARCN1; PPARGC1A; RERE; KHDRBS3; XRN1; RHOBTB3; RPS6KA5; IGF2R; ZNF423; DHH; PHF13; SF3B1; AP2A2; NOVA1; SSRP1; KCNA3 |
| has-miR-196b | CDYL; LRP1B; EPHA7; BACH1; RGL2; NRAS; TRERF1; ZMYND11; CPEB3; COL3A1; SLC31A1; ABCB9; SLC9A6; SNX16; **SMAD6;** HAND1; OSMR; LRRTM3; HOXA5; ZBTB26; UHRF2; HOXB7; PBX3; CBFA2T3; SORCS1; SOCS4; HOXC8; HOXB6; ERG; PDGFRA; CCNJ; OTX1; MAP4K3; GATA6; USP15; ZDHHC21; ING5; SMARCC1; CPD; ZNF281; PPP1R16B; GAN; AQP4; PPP1R15B; COL1A2; CDKN1B; NME4; HMGA2 |
| has-miR-509 | SEC14L5; BCAR1; CRK; TCF7L2; LARP4; VEZF1; CALB1; PLP1; RBMY1F; FCRL5; DEDD; MYPN; ZFAND3; MTSS1; SPATS2L; TRPA1; ENPP2; NLK; NXPH1; TMOD1; CES3; BRDT; ELF3; RAB5C; RBMY1A1; MYOCD; NR1D2; NRN1; COCH; FCAR; CLDN12; KLK7; PBLD; APOD; MEIS1; MAP3K8; MCM8; CEACAM1; OSCAR; RFX5; MLF1; ANKRD34B; CDK17; NETO2; DCUN1D1; ATG7; MMP19; BDH2; NEO1; FRMD5; SHROOM3; RCHY1; NAA30; SLC37A2; PHLPP2; ANO5; WLS; DOCK2; MMGT1; MTMR3; YAP1; MMD2; SERPIND1; ABCB10; BEND6; PCDHA13; BTN3A1; EFCAB1; PCDHA11; SLC2A13; PJA1; PBX3; TRAPPC6B; GPHA2; PTEN; COG5; AMOT; PATL1; HABP4; MARK4; APOBEC2; PCDHA3; WDR5B; TMPRSS11E; MAP3K1; UBR7; ZNF345; DCLK1; PCDHA5; TP53INP1; GTF2I; ARHGAP1; PISD; RBMY1D; PCDHAC1; PRKACB; STAC; PTPN22; NPAS2; RSPH3; IKZF1; KCNJ3; FECH; CEACAM7; ANKRD23; SGCB; CCNJ; ASH2L; DUSP18; SEPSECS; BRWD1; PIK3C2A; TPMT; BTBD7; PPP2R5C; LRRTM1; VGLL4; ATP1B4; B4GALNT2; PDCD6IP; IL1A; DDAH1; KLF6; KIAA0754; DST; PCDHA2; RAC1; SF3B4; USP47; ZIC3; TYRP1; RNF130; TMEM64; TMEM95; SORD; LRRC39; SH3GL3; SNX13; MCFD2; CMTM6; SLC46A1; CTSC; C4orf46; PCDHA6; ANKH; ING1; SYNJ2BP; DMRT3; ERLIN2; PSMB1; EHD3; TRIO; PCDHAC2; ZNF423; PCDHA8; CACNB4; ST8SIA3; C20orf197; PIP5K1B; ACADSB; PCDHA1; PCDHA12; SCG3; ZNF107; UNC13B; ELAVL1; ZNF777; IL13RA1; LAMA5; FBXO27; PCDH8; CUL5; MAGI1; SERPINB9; AHCYL1; PCDHA10; SLC22A4; MAPK1IP1L; GPR158; PCDHA4; OSBP; PCDHA7; PCDHA9; PELO; SFPQ; EPCAM; RNF180; INTS10; USP27X |
| has-miR-326 | RASL10B; VLDLR; RASSF1; PKIA; ELK1; SMAD6; PAX8; RARG; KCNIP2; PPP3CB; GPI; ANKFY1; CORO2B; SEC63; YBX2; LRRTM1; OTP; VPS39; RALGPS2; PALM; C9orf24; DRD2; EPHB3; SLC27A4; TLN1 |
| has-miR-146a | USP3; STRBP; PPP1R11; PTPRA; RARB; ROBO1; SEC23IP; KLF7; SYT1; NUMB; SORT1; JAZF1; IRAK1; PTGFRN; PIP5K1B; TRAF6; NOVA1; SAMD8 |
| has-miR-299-5p | CALU; RSBN1; MAP3K8; MAT2A; PURB; ABR; SIAH1; SOX4; PUM2; RALBP1; SOCS5; CTCF; TCERG1; PPP2R5A; ZDHHC5; HDAC4; GOLGA1; ARIH1 |
| has-miR-520e | ZNF77; TGFBR2; RECK; RUNX2; RGMA; ORMDL3; SYDE1; LARP1B; USP24; NR2E1; WDR20; PBX3; ABCA1; UBE2W; PDE12; TRPS1; AHNAK; KDM1B; TRIM44; LHX8 |
| hsa-miR-138 | CNOT8; PTK2; RELN; BNIP3L; ARHGEF3; ZMYND11; DHDDS; GNAI2; RARA; MACF1; ATP11C; PDE3A; SIRT1; MAP3K11; ANK1; SLC17A7; SIN3A; CALN1; SENP1; PTP4A1; EZH2; SLC20A1; FEM1C; EFNB3; EPHA8; PSMF1; RPS6KA1; KBTBD4; SEMA4C; UNC5D; USP10; NBEA; SOX4; JAZF1; LYPLA1; RHOC; CLNS1A; THAP11; MOCS1; C6orf47; PAPPA; DVL2; TRPS1; USP47; UBP1; TIPARP; PPARGC1A; NEBL; DNAJB6; SFXN2; PDE7B; H3F3B; ARRDC3; TP53INP2; GIT1; LHFPL3; SEH1L; ZNF444; SNCB; PHOX2B; ROCK2 |

TABLE S3: Expression of hsa-miR-196b-3p and hsa-miR-196b-5p in TCGA

| ID | logFC | *P*Value | FDR |
| --- | --- | --- | --- |
| hsa-miR-196b-3p | 3.269789714 | 7.07E-10 | 3.61E-09 |
| hsa-miR-196b-5p | 4.674216894 | 3.55E-24 | 8.44E-23 |
