# Supplementary figures and images for: Construction and Investigation of MicroRNA-mRNA Regulatory Network of Gastric Cancer with Helicobacter pylori Infection
Source: Biochem Res Int. 2020 Jul 25;2020:6285987. doi: 10.1155/2020/6285987 (PMC7410007; doi:10.1155/2020/6285987)

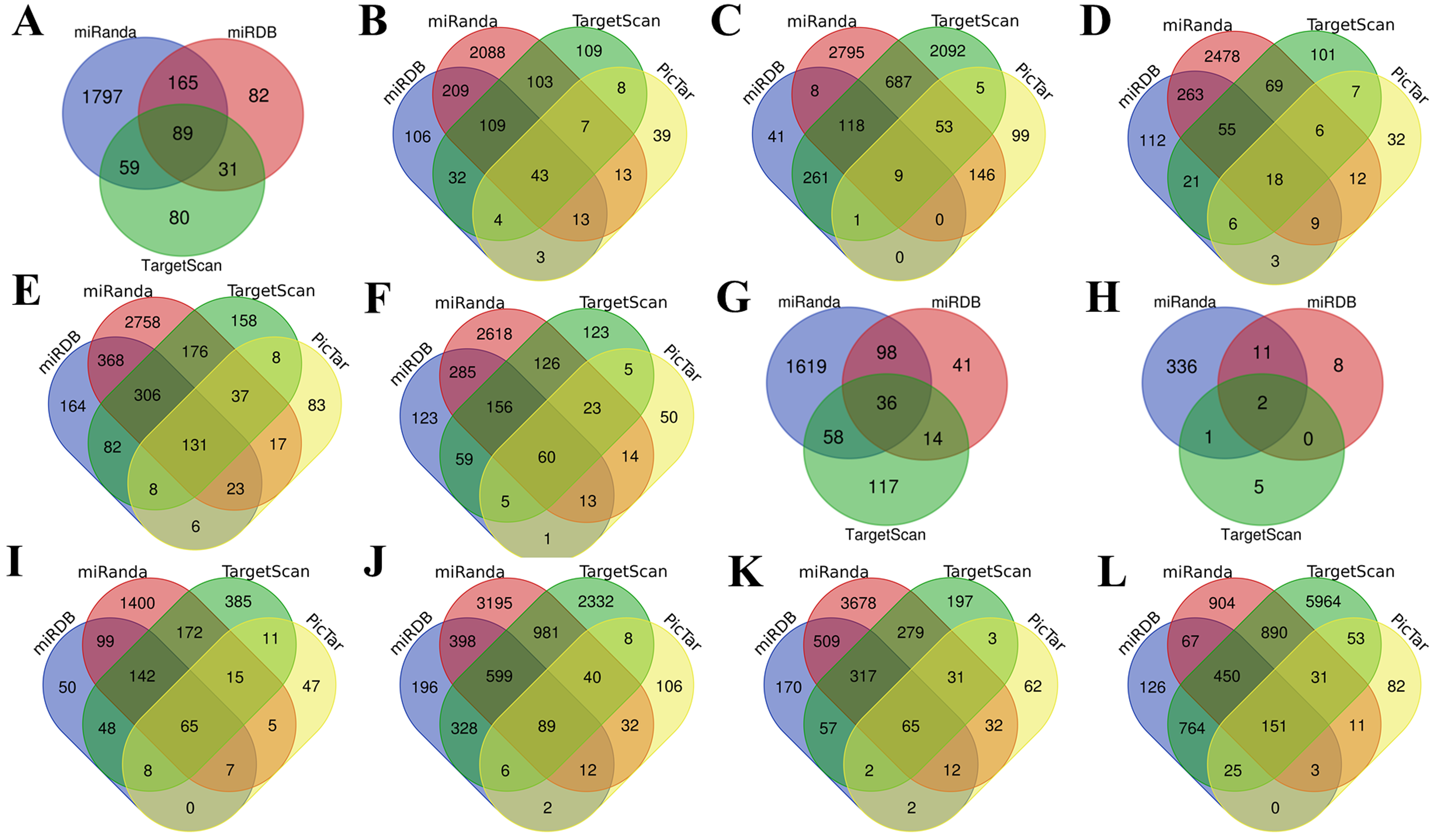

Supplement: Supplementary Materials — Figure S1: Venn diagram of potential targets of DEMs predicted by 4 software programs. Venn for hsa-miR-455 (A), hsa-miR-223 (B), hsa-miR-200a-5p (C), hsa-miR-146b (D), hsa-miR-200a-3p (E), hsa-miR-155 (F), hsa-miR-411 (G), hsa-miR-551b (H), hsa-miR-142-3p (I), hsa-miR-203 (J), hsa-miR-142-5p (K), and hsa-miR-153 (L). Figure S2: Venn diagram of potential targets of DEMs predicted by 4 software programs. Venn for hsa-miR-204 (A), hsa-miR-196b (B), hsa-miR-509 (C), hsa-miR-326 (D), hsa-miR-146a (E), hsa-miR-299-5p (F), hsa-miR-520e (G), and hsa-miR-138 (H). Figure S3: GO and KEGG function analysis of the cross-genes. Circle diagram of (A) GO clusters and (B) KEGG pathway clusters. Table S1: differential expression genes of H. pylori-negative and -positive patients. Table S2: targets of DEMs in the network. Table S3: expression of hsa-miR-196b-3p and hsa-miR-196b-5p in TCGA. [file 6285987.f1.zip › FIGURE S1.tif]

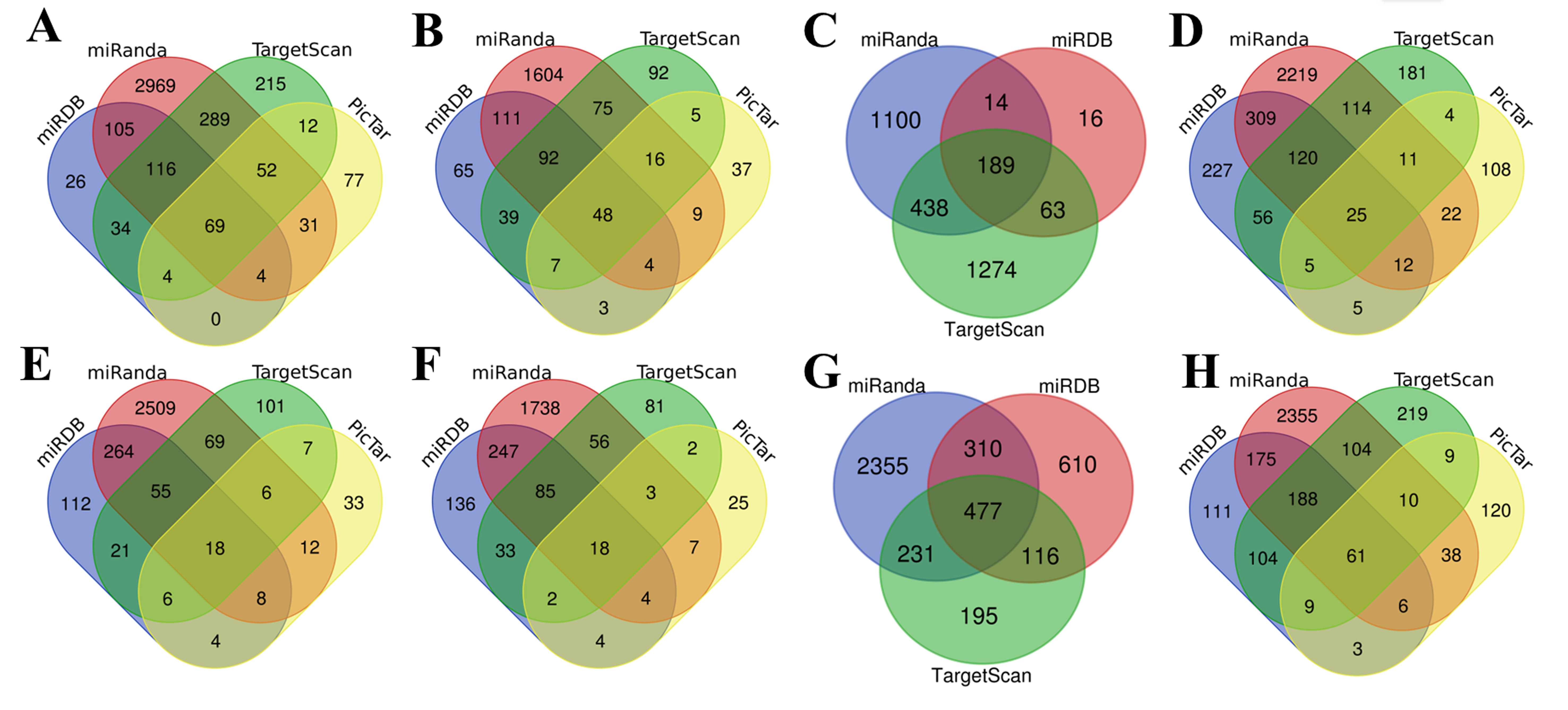

Supplement: Supplementary Materials — Figure S1: Venn diagram of potential targets of DEMs predicted by 4 software programs. Venn for hsa-miR-455 (A), hsa-miR-223 (B), hsa-miR-200a-5p (C), hsa-miR-146b (D), hsa-miR-200a-3p (E), hsa-miR-155 (F), hsa-miR-411 (G), hsa-miR-551b (H), hsa-miR-142-3p (I), hsa-miR-203 (J), hsa-miR-142-5p (K), and hsa-miR-153 (L). Figure S2: Venn diagram of potential targets of DEMs predicted by 4 software programs. Venn for hsa-miR-204 (A), hsa-miR-196b (B), hsa-miR-509 (C), hsa-miR-326 (D), hsa-miR-146a (E), hsa-miR-299-5p (F), hsa-miR-520e (G), and hsa-miR-138 (H). Figure S3: GO and KEGG function analysis of the cross-genes. Circle diagram of (A) GO clusters and (B) KEGG pathway clusters. Table S1: differential expression genes of H. pylori-negative and -positive patients. Table S2: targets of DEMs in the network. Table S3: expression of hsa-miR-196b-3p and hsa-miR-196b-5p in TCGA. [file 6285987.f1.zip › FIGURE S2.tif]

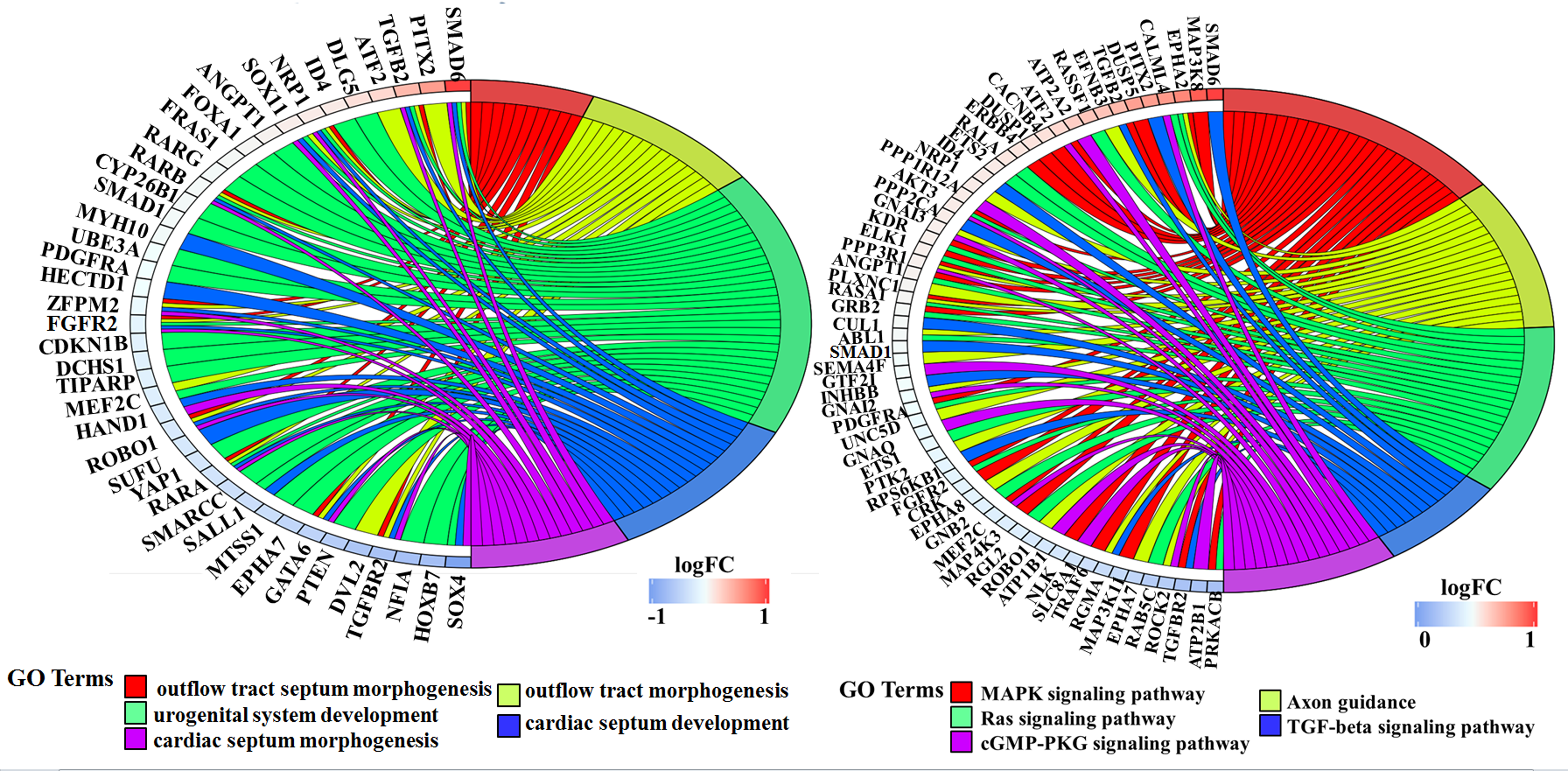

Supplement: Supplementary Materials — Figure S1: Venn diagram of potential targets of DEMs predicted by 4 software programs. Venn for hsa-miR-455 (A), hsa-miR-223 (B), hsa-miR-200a-5p (C), hsa-miR-146b (D), hsa-miR-200a-3p (E), hsa-miR-155 (F), hsa-miR-411 (G), hsa-miR-551b (H), hsa-miR-142-3p (I), hsa-miR-203 (J), hsa-miR-142-5p (K), and hsa-miR-153 (L). Figure S2: Venn diagram of potential targets of DEMs predicted by 4 software programs. Venn for hsa-miR-204 (A), hsa-miR-196b (B), hsa-miR-509 (C), hsa-miR-326 (D), hsa-miR-146a (E), hsa-miR-299-5p (F), hsa-miR-520e (G), and hsa-miR-138 (H). Figure S3: GO and KEGG function analysis of the cross-genes. Circle diagram of (A) GO clusters and (B) KEGG pathway clusters. Table S1: differential expression genes of H. pylori-negative and -positive patients. Table S2: targets of DEMs in the network. Table S3: expression of hsa-miR-196b-3p and hsa-miR-196b-5p in TCGA. [file 6285987.f1.zip › FIGURE S3.png]
